# A New Remedy in Cystitis and Lithiasis—Pichi (Fabiana Imbricata)

**Published:** 1886-06

**Authors:** Hal. C. Wyman


### A New Remedy in Cystitis and Lithiasis—Pichi (Fabiana Imbricata).

By Hal. C. Wyman, M. D.

So short a time has elapsed since this remedy was offered to the medical profession that a report on its use may seem premature. But so long as the report is confined to a simple statement of facts, leaving the reader to judge of the influence the medicine may have had in bringing about the results, no one need scruple at the evidence.

The first case in which I used the fl. ext. pichi was early in January, when my attention was called to the drug by Dr. J. E. Clerk, the chemist of this city. A Mr. G., aged 23 years, who contracted gonorrhœa a year or more ago, and who had stricture of the urethra, which I found necessary to divulse a few days before Christmas. A copious hemorrhage from the deep urethra followed the operation, and a few days later severe vesical tenesmus disturbed the patient so that sleep and appetite vanished. All the symptoms of typical cystitis speedily developed. Morphine, belladonna, hyoscyamus and alkalies were used internally and by the rectum for a week without materially modifying the symptoms. So irritable did the bladder become that it would not retain urine longer than five to fifteen minutes. I now began the use of the fluid extract of pichi in 15-drop doses in water once in three hours. The tenesmus and pain began to decline after twenty-four hours, the mucus and pus also to diminish in quantity, and, at the end of the week, the bladder would retain its contents three hours without causing the patient any inconvenience.

This patient has now, March 8th, fully recovered. I think the cystitis was of traumatic origin—the harsh manipulation of the vesical mucous membrane with the divulsing apparatus. Thompson's instrument was the one used.

Mrs. ———, aged 27, had chronic cystitis, due to neglected retention of urine, following accouchement. She had overflow before the catheter was used to relieve her. I found it necessary to divulse her urethra to insure thorough evacuation of her bladder. It was very irritable after the operation. Pichi, combined with fluid ext. hyoscyamus, controlled the painful symptoms, and gave the bladder the physiological rest essential to a cure.

Mr. H., aged 71 years, had been getting up nights to pass urine for the last five years. Ten months ago he found he could not empty his bladder, and was compelled to call a surgeon to use the catheter. A severe chill followed, and he was very sick for two months, having the catheter used every day. He learned to use it himself, and continued to use it daily until last February, when he had another chill, and his left testicle swelled and inflamed. He was too sick to use the instrument. I was summoned by his medical attendant in consultation, and opened a large abscess of the left testicle, and advised against further use of the catheter. His prostate was greatly enlarged, Opium and camphor suppositories relaxed the spasm of the prostatic muscles so that he got along without the catheter. His urine was heavily loaded with urates. Mucus, blood and pus were present in abundance. He was put on fluid ext. pichi, and continued its use for three weeks. He has made an excellent recovery.

I might enumerate numbers of cases of lumbago and sciatica in the course of which urates were precipitated from the urine in large quantities, and which recovered while the patient was taking the pichi. Combined with a potassium salt, I have found it to act more quickly than any other remedy in bringing about a solution of the urates and relieving the rheumatic neuralgia so frequently associated with that unstable condition known as lithuria, phosphatism, etc. A formula I have often used is—

| | | |
|---|---|---|
| ℞ Fl. ext. pichi, | - - - - | ℥i. |
| Potass. nitrate, | - - - - | ʒi. |
| Simple elixir, | - - - - | ℥iii. M. |

S.—Teaspoonful once in two hours.

—*Therapeutic Gaz.*

